# Critical physicochemical and biological attributes of nanoemulsions for pulmonary delivery of rifampicin by nebulization technique in tuberculosis treatment

**DOI:** 10.1080/10717544.2017.1384298

**Published:** 2017-10-24

**Authors:** Kifayatullah Shah, Lai Wah Chan, Tin Wui Wong

**Affiliations:** a Non-Destructive Biomedical and Pharmaceutical Research Centre, iPROMISE, Selangor, Malaysia;; b Particle Design Research Group, Faculty of Pharmacy, Universiti Teknologi MARA, Selangor, Malaysia;; c Department of Pharmacy, Faculty of Science, National University of Singapore, Republic of Singapore

**Keywords:** Aerosolization, chitosan, chitosan-folate, inhalation, nanoemulsion

## Abstract

The study investigated aerosolization, pulmonary inhalation, intracellular trafficking potential in macrophages and pharmacokinetics profiles of rifampicin-oleic acid first-generation nanoemulsion and its respective chitosan- and chitosan-folate conjugate-decorated second and third-generation nanoemulsions, delivered via nebulization technique. The nanoemulsions were prepared by conjugate synthesis and spontaneous emulsification techniques. They were subjected to physicochemical, drug release, aerosolization, inhalation, cell culture and pharmacokinetics analysis. The nanoemulsions had average droplet sizes of 40–60 nm, with narrow polydispersity indices. They exhibited desirable pH, surface tension, viscosity, refractive index, density and viscosity attributes for pulmonary rifampicin administration. All nanoemulsions demonstrated more than 95% aerosol output and inhalation efficiency greater than 75%. The aerosol output, aerosolized and inhaled fine particle fractions were primarily governed by the size and surface tension of nanoemulsions in an inverse relationship. The nanoemulsions were found to be safe with third-generation nanoemulsion exhibiting higher cell internalization potential, reduced plasma drug concentration, and higher lung drug content.

## 1. Introduction

Tuberculosis is a major global health challenge. It is the second leading cause of death from an infectious disease worldwide after human immunodeficiency virus (Pham et al., 2015). The disease mostly affects the lung and remains localized in 75–80% of the cases, but can also disseminate to other areas causing extrapulmonary tuberculosis (Yadav et al., 2009; Sosnik et al., 2010). The current standard treatment for pulmonary tuberculosis includes a multi-drug regimen of four first-line drugs (isoniazid, rifampicin, pyrazinamide and ethambutol) for two months, followed by a subsequent continuous phase of four months with rifampicin and isoniazid (Blasi et al., 2009; WHO, 2010), with ethambutol replaced by streptomycin in patients suffering from *Tuberculous meningitis* and addition of ethambutol to the continuous phase treatment in isoniazid-resistant patients. The treatment is effective in both human immunodeficiency virus-infected and un-infected persons, and the success rate is at least 85% (WHO, 2013). However, the efficacy of anti-tubercular therapy is limited by constraints of drug dose, adverse drug effects and inadequate drug penetration into pathological sites because these lesions are poorly vascularized (Pham et al., 2015). It leads to poor patient compliance and multi-drug resistant tuberculosis. The poor patient compliance is the major cause of drug resistance (Bagchi et al., 2010), as a function of traveling expenses to treatment centers, poor patient information and communication, male gender, alcoholism and homelessness.

Rifampicin (C_43_H_58_N_4_O_12_) is a high molecular weight borderline class II drug, of which its rate and extent of dissolution are critical for obtaining optimum bioavailability (Amidon et al., 1995). It is effective as an antibiotic both in extracellular and intracellular environment of the lung (Friedman & Selwyn, 2001). However, chronic oral or intravenous therapies cause systemic side effects, such as rash, fever, gastrointestinal disturbances, jaundice and immunological reactions leading to poor patient compliance. In tuberculosis, the bacilli survive quite successfully in phagocytes and can also replicate in phagosomes of alveolar macrophages (Muttil et al., 2009). Therefore, it could be interesting not only to aim for high antibiotic concentration at the lung epithelium, but also to specifically target the alveolar macrophages harboring mycobacteria. Macrophages possess numerous surface receptors that involve sugar recognition mechanism. These receptors recognize the corresponding sugar on non-reducing terminal of sugar chains where cellular uptake is then mediated (Wadhwa & Rice., 1995). The success of selective drug delivery to macrophages is crucially dependent on the ability to develop ligand-decked carrier system which can lead to accumulation of drug in the target cells and exclude non-target cells.

Chitosan consists of glucosamine and N-acetylglucosamine units and it is a well-known ligand for mannose receptors of macrophages harboring mycobacteria due to its N-acetylglucosamine residue (Peluso et al., 1994; Feng et al., 2004). The chitosan is a biocompatible and biodegradable polymer (Aam et al., 2010; Baldrick, 2010) that has antimicrobial activities against bacteria, virus and fungi (Jumaa et al., 2002; Aam et al., 2010; Bernkop-Schnürch & Dünnhaupt, 2012). It provides synergistic effects in combination with antibiotics, and thus can be used to enhance the antimicrobial activity of pharmaceuticals (Tin et al., 2009). Chitosan has been conjugated with ligands, such as folate, for which receptors are over-expressed on the activated macrophages (Rollett et al., 2012; Puligujja et al., 2015), aiming to provide excellent opportunity for targeted drug delivery.

Direct delivery of anti-tubercular drugs to the lungs is an interesting strategy to maximize drug concentration at the target site, while reducing the systemic drug exposure and possible development of drug-resistant bacterial strains (Doan et al., 2011). Various inhalation systems have been developed for tuberculosis treatment, such as polymeric microparticles (O’Hara & Hickey, 2000; Suarez et al., 2001; Tomoda & Makino, 2007; Coowanitwong et al., 2008; Ohashi et al., 2009; Doan et al., 2011; Park et al., 2014), solid lipid microparticles (Maretti et al., 2014), polymeric and solid lipid nanoparticles (Sung et al., 2009; Booysen et al., 2013; Chuan et al., 2013), with the aim to primarily target the infected alveolar macrophages and to some extent local lung tissue. Some authors focus on producing pure drug powders without excipients to ensure maximum drug loading (>75% drug), that target lung epithelial tissue and systemic circulation (Son & McConville, 2011; Chan et al., 2013). The dry powder formulations suffer problems in powder processing (poor flowabilty from dry powder inhaler devices) and re-dispersion (strong agglomeration and adhesion) due to the small size and large specific area of contact between the engineered particles, making the powder unsuitable for efficient aerosolization and inhalation (Weers, 2000).

Among liquid dispersions encapsulating anti-tubercular drugs delivered in the form of an aerosol, the pressurized packed liposomal systems are well documented (Farr et al., 1987; Vyas & Sakthivel, 1994). Nebulization of such liposomal dispersion has been shown to allow drug penetration into the peripheral region of the lung. Similar promising results have been found in previous studies, where liposomal antibiotic formulations are administered by inhalation to improve the drug efficacy against the tuberculosis in BALB/c mice and male albino rats of Sprague–Dawley and Wistar origin (Kurunov et al., 1994; Vyas et al., 2004; Chono et al., 2008). Like liposomes, the niosomes (Sing et al., 2011) and dendrimers (Kumar et al., 2006) are also found to be effective in reducing drug dosage and dosing frequency.

The aforementioned liquid carrier systems suffer from the problems of low drug encapsulation, burst drug release and short drug half-life (Pandey & Khuller, 2005). The nanoemulsion possesses a solution-like nature as well as high drug solubilizing and protection properties (Amani et al., 2010). Owing to reduced particle size (< 100 nm), it is deemed able to improve the drug bioavailability through facilitating mucosal penetration of drug (Mason et al., 2006). The nanoemulsion can be designed as a targeted drug carrier through decorating the droplets’ surfaces with targeting ligands. Lately, it has been reported that the aerosolization and inhalation performances of nanoemulsions are distinctively superior to those of commercial suspensions and related formulations, such as liposomes, micelles and nanoparticles (Amani et al., 2010; Laouini et al., 2014). However, the physicochemical characteristics of a nanoemulsion responsible for its improved performance in inhalational drug delivery are yet to be investigated.

The current study aimed to design first-generation nanoemulsion that was decorated with oligochitosan or oligochitosan-folate conjugate as the targeting ligands of macrophages in the development of second and third-generation nanoemulsions, respectively. The aerosolization and inhalation performances of these nanoemulsions were evaluated as a function of the physicochemical characteristics of the nanoemulsions with the aim to identify critical dosage form properties in pulmonary rifampicin delivery. In addition, the cytotoxicity profiles and intracellular trafficking potential of the nanoemulsions in macrophages as well as the pharmacokinetics profiles of rifampicin in animal model were evaluated.

## 2. Materials and methods

### 2.1. Materials

Rifampicin (Taizhuo Tianrui Pharmaceutical Co. Ltd., China) was chosen as a drug of interest. Oleic acid (Fluka, Seelze, Germany) was employed as an oil phase to dissolve rifampicin. Tween 80 (Fisher Scientific, Loughborough, UK) was selected as an emulsifier and ethanol 96% (Analytical grade; Merck, Darmstadt, Germany) was used as a drug co-solubilizer. Sodium chloride (Merck, Darmstadt, Germany) was used to prepare normal saline which constituted the aqueous phase. Chitosan oligosaccharide lactate (Sigma-Aldrich, St. Louis, MO) was employed as mucoadhesive, bioadhesive and targeting moiety for macrophages. Folic acid (Sigma-Aldrich, St. Louis, MO) was used as targeting ligand of macrophage. 1-(-Dimethyl aminopropyl)-3-ethylcarbodiimide) hydrochloride (EDC; Acros Organics, Morris Plains, NJ) was used as a coupling agent in conjugation of folic acid to oligochitosan. Sodium hydroxide (Merck, Darmstadt, Germany), potassium dihydrogen phosphate (Merck, Darmstadt, Germany) and l(+)-ascorbic acid (HmbG Chemicals, Hamburg, Germany) were used as solvent component, pH modifier or buffer constituent and as preservative, respectively.

Alveolar macrophages NR8383 (CRL-2192TM, ATCC^®^, Manassas, VA) was the cell line of interest. The cell line was cultured using Ham’s F-12 K medium (Sigma-Aldrich) enriched with fetal bovine serum (GibcoTM, Invitrogen, South America), 200 mM l-glutamine (GibcoTM Invitrogen, Brazil) and gentamicin (GibcoTM Invitrogen, China). Trypsin-EDTA 0.025% (GibcoTM Invitrogen, Canada) was used to detach cells from the flask during collection, with phosphate buffer saline (Sigma Aldrich, St. Louis, MO) as the cleaning liquid. The cells were cryopreserved in a mixture of 5% dimethyl sulfoxide and 95% Ham’s F-12 K medium containing 1% 200 mM l-glutamine, 15% fetal bovine serum and 1% gentamicin. The fluorescein isothiocynate (FITC; Acros Organics, Morris Plains, NJ) was used as tagging agent and 3-(4, 5-dimethylthiazol-2-yl)-2, 5-diphenyltetrazolium bromide (MTT; Sigma Aldrich, Hamburg, Germany) was used in assessment of cell viability.

### 2.2. Synthesis of chitosan-folate conjugate

Synthesis of chitosan-folate conjugate was attempted according to the method reported by Li et al. (2011) with slight modifications. Briefly, 0.2 g of oligochitosan was dissolved in 40 ml of de-ionized water. Both folic acid (0.04 g) and EDC (0.04 g) were dissolved in 10 ml of anhydrous dimethyl sulfoxide (Fisher Scientific, Loughborough, UK) to form a solution, which was added dropwise into freshly prepared oligochitosan solution and stirred for 24 h in the dark at 25 ± 1 °C. The resultant product was coagulated by adding 300 ml of acetone (Merck, Darmstadt, Germany) and centrifuged (PK121 R, ALC, Princeton, NJ) at 3400 rpm for 5 min. The supernatant was discarded and the solid residue was collected. The solid residue was then purified by dialysis against distilled water for 2 days using a dialysis membrane (molecular weight cutoff: 1000 Da; Spectra/Por, Spectrum laboratories, Rancho Dominguez, CA). The resultant yellow colored chitosan-folate conjugate was collected and freeze dried at –50 °C for 24 h. The freeze dried samples were kept in a desiccator until use.

### 2.3. Characterization of chitosan-folate conjugate

#### 2.3.1. Fourier-transform infrared (FTIR) spectroscopy

Accurately weighed 2 mg each of oligochitosan, folic acid and lyophilized chitosan-folate conjugate was mixed with 78 mg of dry potassium bromide powder (FTIR grade; Aldrich, Munich, Germany), respectively, and the mixture was ground into a fine powder using an agate mortar. The fine powder was then compressed into a disc. It was scanned at a resolution of 4 cm^−1^ over a wavenumber region of 400 to 4000 cm^−1^ using a FTIR spectrometer (Spectrum RX1 FTIR system, PerkinElmer, Norwalk, CT). The characteristic peaks of IR transmission spectra were recorded. At least triplicates were carried out for each batch of samples and the results averaged.

#### 2.3.2. Nuclear magnetic resonance (NMR) spectroscopy

^1^H-NMR spectra of folic acid, oligochitosan and chitosan-folate conjugate were recorded using ultra shield plus 500 nuclear magnetic resonance spectrometer (Bruker, Rheinstetten, Germany) with deuterated dimethyl sulfoxide as the solvent for folic acid, while oligochitosan and chitosan-folate conjugate were dissolved in 1% deuterated acetic acid before analysis. The clear solutions were then introduced into NMR tubes for analysis. At least triplicates were carried out for each batch of samples.

#### 2.3.3. Folate content

The content of folate in chitosan-folate conjugate was determined by dissolving the conjugate in 0.01 N hydrochloric acid solution (pH = 3) at the concentration of 0.2 mg/ml under continuous magnetic stirring at 40 °C for 24 h. The pH of the solution was adjusted to 10 by 2 N sodium hydroxide solution (200 µl) in order to dissolve the folate and to precipitate the oligochitosan. The resultant mixture was then filtered, and the filtrate was analyzed at 365 nm using the UV-visible spectrophotometer (Cary 50 Conc, Varian Australia Pty. Ltd., Mulgrave, Australia) with chitosan solution as the control sample. The results were taken as an average of three readings.

### 2.4. Preparation of nanoemulsions

Rifampicin-loaded first generation oil-in-water (o/w) nanoemulsion was prepared by spontaneous emulsification method using oleic acid, ethanol, Tween 80 and normal saline as oil, drug co-solubilizer, surfactant and aqueous phase, respectively. The second and third-generation nanoemulsions were prepared by decorating the oil droplets of the first-generation nanoemulsion with chitosan or chitosan-folate conjugate to introduce targeting ability for alveolar macrophages.

#### 2.4.1. First-generation nanoemulsion

Briefly, the oil phase was prepared by dissolving 175 mg of rifampicin (0.875% w/w) in 1 g (5% w/w) of oleic acid with the addition of variable amounts, i.e. 1% (0.2 g), 5% (1 g) and 10% w/w (2 g) of ethanol as drug co-solubilizing agent under continuous magnetic stirring at 600 rpm for 2 h (Table 1). Normal saline was prepared by dissolving 0.9 g of sodium chloride in a sufficient quantity of de-ionized water to produce a final volume of 100 ml, and filtered through filter paper (Whatman Ltd., Kent, UK). The aqueous phase was prepared by dissolving variable amounts i.e. 5% (1 g), 10% (2 g) and 15% w/w (3 g) of Tween 80 in a specified quantity of normal saline under constant magnetic stirring at 600 rpm for 2 h (Table 1). The oil phase was added drop-wise to the aqueous phase in the presence of continuous magnetic stirring at the same speed and at 25 ± 0.1 °C to produce 20 g of first-generation nanoemulsion. The formed nanoemulsion was homogenized at 24,000 rpm (Ultra Turrax T10, IKA, Staufen, Germany) for 10 min.

**Table 1.** Composition of the nanoemulsion formulations.

Composition (% w/w)									
	First-generation nanoemulsion					Second-generation nanoemulsion			Third-generation nanoemulsion
	L1	L2	L3	L4	L5	L6	L7	L8	L9
Oleic acid	5.00	5.00	5.00	5.00	5.00	5.00	5.00	5.00	5.00
Rifampicin	0.88	0.88	0.88	0.88	0.88	0.88	0.88	0.88	0.88
Ethanol	1.00	1.00	1.00	5.00	10.00	1.00	1.00	1.00	1.00
Tween-80	5.00	10.00	15.00	15.00	15.00	15.00	15.00	15.00	15.00
Chitosan	0.00	0.00	0.00	0.00	0.00	0.10	0.25	0.50	0.00
Chitosan-folate conjugate	0.00	0.00	0.00	0.00	0.00	0.00	0.00	0.00	0.25
Normal saline	88.13	83.13	78.13	74.13	69.13	78.03	77.88	77.63	77.88

#### 2.4.2. Second and third-generation nanoemulsions

The selected first-generation nanoemulsion was further decorated for targeted drug delivery to alveolar macrophages through adding different quantities of oligochitosan (0.1, 0.25 and 0.5% w/w of the second generation nanoemulsion) or oligochitosan-folate conjugate (0.25% of the third-generation nanoemulsion) as targeting ligands (Table 1). Briefly, the oligochitosan or oligochitosan-folate conjugate was added into the aqueous phase prior reacting with the organic phase. The first, second and third-generation nanoemulsions were subjected to physicochemical characterization and had their aerosolization and inhalation performances evaluated.

### 2.5. Physicochemical characterization of nanoemulsions

#### 2.5.1. Size and size distribution

The mean droplet size (Z-average) and size distribution (PDI) of the nanoemulsions were measured using photon correlation spectroscopy with Malvern Zetasizer Nano ZS 90 (Malvern Instruments Ltd., Worcestershire, UK) at 25 ± 0.1 °C in quartz cell with a detect angle of 90°. To avoid multiple scattering effects, the samples were diluted (10 ×) with normal saline prior to particle size measurements. Results were taken as an average of at least three readings.

#### 2.5.2. Zeta potential

The zeta potential of nanoemulsions was measured using photon correlation spectroscopy with Malvern Zetasizer Nano ZS 90 (Malvern Instruments Ltd., Worcestershire, UK) at 25 ± 1 °C in zeta potential cell with a detect angle of 90°. Each nanoemulsion sample in a quantity of 700 µl was loaded in the folded capillary cell integrated with gold electrode. Three measurements were conducted and the results averaged.

#### 2.5.3. pH

The pH of nanoemulsions was evaluated in triplicates using a calibrated digital pH meter (Mettler Toledo, Greinfesee, Switzerland) at 25 ± 1 °C.

#### 2.5.4. Viscosity

The specific viscosity of the nanoemulsions was measured by loading the sample in the capillary tube viscometer type B (Poulten selfe & Lee Ltd., Essex, UK) at 37 ± 0.5 °C. The sample was allowed to flow between two points and the time taken by the sample to flow was recorded. The normal saline was used as the control. The specific viscosity was calculated using the following equation:

(1) $$\text{Specific viscosity}\text{=}\frac{\text{flow time of sample }–\text{ flow time of solvent}}{\text{flow time of solvent}}$$

At least triplicates were carried out for each sample and the results averaged.

#### 2.5.5. Surface tension

The surface tension of nanoemulsions was determined using a laboratory K-6 tensiometer (KRUSS GmbH, Hamburg, Germany) equipped with a ring at 25 ± 1 °C. At least triplicates were carried out for each sample and the results averaged.

#### 2.5.6. Density

The density of nanoemulsions was determined using Anton Paar type DMA35N, densitometer (Anton Paar GmbH, Graz, Austria) at 25 ± 1 °C. Results were taken as an average of three readings.

#### 2.5.7. Refractive index

The refractive index, being an optical property, can be used to characterize the isotropic nature of the nanoemulsion. The refractive index of nanoemulsions was determined using the liquid digital refractometer (Abbe Max AMAX20, MISCO, Cleveland, OH) at 25 ± 1 °C. Triplicates were carried out and the result averaged.

#### 2.5.8. Morphology

The structure of the primary nanoemulsion was visualized using transmission electron microscopy technique (TEM; Tecnai G2 20 S, TWIN, FEI, Eindhoven, Netherland). A drop of nanoemulsion was placed on a 400 mesh carbon-coated copper grid where it was rapidly adsorbed. The excessive liquid was removed using a blotting paper, and the sample was treated with 2% uranyl acetate (Electron Microscopy Sciences, Hatfield, PA) for 10 min, after which it was washed with warm water and left to dry before subjecting to microscopic viewing at a current voltage of 200 kV.

The surface morphology of nanoemulsion droplets was also examined using environmental scanning electron microscopy technique (ESEM; Quanta FEG 450, FEI, Eindhoven, Netherland). The nanoemulsion was first centrifuged at 10,000 rpm for 10 min to separate the aqueous phase from dispersed oil droplets. The supernatant was removed, while the nanoemulsion droplets were obtained as sediment. The sediment was treated with 2 to 3 drops of osmium tetra-oxide (Fluka Analytical, USA) for 2 h, diluted with 0.1 M phosphate buffer for fixation. The sample was vortexed to disperse the oil droplets and centrifuged again at 10,000 rpm for 10 min to remove the fixation medium. The supernatant was removed while the sediment pellet of oil droplets was washed three times with 1 ml of 0.1 M phosphate buffer with repeated cycles of centrifugation at the same speed for the same duration at 25 ± 1 °C while dispersing the oil droplets pellet each time after centrifugation. Finally the sample was dehydrated with acetone and a drop of nanoemulsion was placed on a carbon film on 400 mesh copper grid, which was located on a liquid sample holder and representative sections were photographed at an accelerating voltage of 20 kV using gaseous secondary electron detector. The roughness of nanoemulsion droplets was computed from the micrographs using a processing software image J (NiH, USA).

#### 2.5.9. *In vitro* drug release

The drug release profiles of first, second and third-generation nanoemulsions were determined using USP pH 7.4 buffer as the simulated dissolution medium for the lung. An accurately weighed amount of sample was encapsulated in a hard gelatin capsules (size ‘0’) for ease of sample introduction to the dissolution medium, and placed in 500 ml of phosphate buffer pH 7.4 under sink condition at 37 ± 0.2 °C. It was agitated at 50 strokes/min in a shaker bath (Memmert, Osterode, Germany). Aliquots of 5 ml were withdrawn at predetermined time intervals, filtered using 0.45 µm syringe filter and assayed spectrophotometrically for rifampicin content at 475 nm (Cary 50 Conc, Varian Australia Pty. Ltd., Mulgrave, Australia) using similarly processed blank nanoemulsion as control. The percentage of drug released was calculated with respect to the total drug content of the nanoemulsion. Each experiment was carried out in triplicates and the results averaged.

Where applicable, the mechanism of drug release was investigated by fitting the drug release data into Korsmeyer–Peppas dissolution model as expressed by

(2) $$F=Kt^{n}$$

where *F* indicates fractional drug released into the dissolution media at time *t* (min), *K* is the drug release rate constant and *n* is the diffusion exponent indicative of drug release mechanism. The *n* and *K* values were obtained from the plots of log *F* against log *t,* with the goodness of fit of the drug release data being evaluated by linear regression. The value of *n* ≤ 0.45 suggests Fickian diffusional (case I) release, 0.45 < *n* < 0.89 suggests non-Fickian (anomalous) release, *n* = 0.89 represents case II (zero-order) release, and *n* > 0.89 represents super case II release.

### 2.6. Aerosolization and inhalation study

#### 2.6.1. Total aerosol output and aerosol output rate

Three milliliters of each nanoemulsion was loaded into the medication compartment of the Omron nebulizer (OMRON Healthcare Co. Ltd., Kyoto, Japan), which was horizontally positioned. The reservoir and mouthpiece of the Omron nebulizer were both parallel to the ground. The nebulization was commenced to dryness and the required time was determined. Total aerosol output was determined gravimetrically by measuring the weight of nebulizer before and after nebulization, while output rate was calculated by dividing the weight of nanoemulsion nebulized by the time of nebulization and expressed as g/min. At least triplicates were carried out for each sample and the results averaged.

#### 2.6.2. Laser diffraction analysis

The aerodynamic and inhalation characteristics of the nebulized nanoemulsions were evaluated using the laser diffraction method. For non-volatile nebulized aerosols, findings by laser diffraction correlate well with lung deposition *in vivo* (Clark, 1995). The droplet size distribution measurements of nanoemulsions were conducted in triplicates using a real-time sizing instrument Malvern Spraytech (Malvern Instruments Ltd., Worcestershire, UK) equipped with RT Sizer Software. The nanoemulsion was loaded into a nebulizer. The nebulizer was held perpendicularly to the laser lens line of the instrument at a distance of ∼2 cm from the laser beam. The air containing the generated aerosol was directed as a well-defined stream through the laser beam of the particle size analyzer. The nanoemulsions were nebulized at a flow rate of 15 l/min and the aerosol was aspirated with the help of a suction pump on the opposite side to draw the aerosol through the laser beam for droplet size analysis. The volume median diameter (Dv(50); 50% undersize), particle diameter at 10% of the undersized particle distribution curve Dv(10), particle diameter at 90% of the undersized particle distribution curve Dv(90), specific surface area (SSA), surface area weighted mean diameter (D[3,2]), volume weighted mean diameter (D[4,3]), fine particle fraction (FPF) at two levels (FPF_<3 µm_ and FPF_<5 µm_) and span of inhalation droplets were determined. The results were taken as an average of three experiments.

#### 2.6.3. Cascade impactor analysis

The aerodynamic and inhalation characteristics of the nanoemulsions were measured using an eight stage Anderson cascade impactor (ACI; Copley Scientific Ltd, Nottingham, UK), that allowed chemical assay of the active pharmaceutical ingredient in the deposited aerosol. The ACI was first assembled from filter to stage ‘0’ with attached induction port that was clamped and sealed together with FDA-approved silicone rubber O-rings (Copley Scientific Ltd, Nottingham, UK) to prevent inter-stage leak. The ACI was connected at the other end to a vacuum source. Each of the nanoemulsion was loaded in the medication compartment of an OMRON MicroAIR U22 nebulizer (OMRON Healthcare Co. Ltd., Kyoto, Japan) and nebulized into the cascade impactor operated at a flow rate of 15 l/min (typical for the mid-inhalation flow rate of a healthy adult or sedentary inspiratory flow rate). Nebulization was commenced to dryness on continuous mode. Following dosage emission, each plate of ACI along with mouth piece, induction port and filter was washed with phosphate buffer pH 7.4 into separate glass scintillation vials. The respective rifampicin content of the washing solutions was then assayed spectrophotometrically at 475 nm.

The cumulative particle size distribution functions obtained from the ACI were plotted on log probability graph. The mass median aerodynamic diameter (MMAD) was calculated as the particle size at the 50th percentile on the graph. The geometric standard deviation (GSD), representing the spread of aerodynamic particle size distribution, was calculated as the square root of the ratio of particle size at the 84.13th percentile to the 15.87th^h^ percentile. Emitted dose (ED) depicted the sum of rifampicin content collected from mouthpiece, induction port, all stages and the filter. Deposited dose (DD) was the sum of rifampicin content deposited on all stages (0–7) and the filter. Percent dispersed (PD) and percent inhaled (PI) expressed the percentage of ED and DD based on the total dose (TD) as described by Equations 3 and 4:

(3) Percent dispersed=ED/TD×100

(4) Percent inhaled=DD/TD×100

The fine particle dose (FPD) was calculated at two levels, i.e. FPD_<5 µm_ and FPD_<3 µm_ as the dose deposited on stage 3 to filter and stage 4 to filter, respectively, generally representing the drug deposition in the deep and peripheral lung. The FPF and respirable fraction (RF) were expressed as the percentage of FPD to ED and DD, respectively, as shown in Equations 5 and 6:

(5) Fine particle fraction=FPD/ED

(6) Respirable fraction=FPD/DD

All aerosolization and inhalation parameters were expressed as mean ± standard deviations from triplicates experiments.

### 2.7. Intracellular trafficking

The alveolar macrophages NR8383 cell lines (1.5 × 10^4^ cells/ml) were seeded in 25 cm^2^ flask (Nunclon, Thermo Fisher Scientific, Roskilde, Denmark) containing 5 ml of complete Ham’s F-12 K medium enriched with 1% of 200 mM l-glutamine, 15% of fetal bovine serum and 1% gentamicin as essential amino acid, essential nutrients and antibiotic, respectively. It was incubated at 37 °C in an atmosphere with 5% carbon dioxide and 95% relative humidity. The medium of the cell culture was replaced twice weekly, and the cells were observed under inverted microscope (Leica DMI4000 B, Leica Microsystem, Wetzlar, Germany) for their state of confluence. On day 6 or when the confluence was ≥80%, the complete medium was discarded. The cell line was washed with 2 ml of phosphate buffer saline to remove the serum that could possibly interfere with the subsequent cell detachment process of trypsin-EDTA. The washed cell line was incubated with 1 to 1.5 ml of 0.025% trypsin-EDTA for 2 min and tapped to detach cells from the inner flask surfaces. Two milliliters of complete media was subsequently added into the flask. The suspension of cells was collected and sub-cultured into a new flask. Cells between passages 3 to 15 were used for the subsequent experiments.

For cellular uptake experiments, 2 ml of cell suspension was transferred to Glass Bottom CELLview™ culture dishes (Greiner Bio-one, Frickenhausen, Germany) and allowed to adhere overnight. The medium was removed, and the cells were washed twice with phosphate buffer saline (pH 7.4) to remove the remnant growth medium. The cells were subsequently treated with 1 ml of phosphate buffer saline containing FITC-labeled chitosan- (second generation, L7) and chitosan-folate conjugate-decorated (third generation, L9) nanoemulsions at a concentration of 0.1 g/ml. The cells were incubated at 37 °C under 5% carbon dioxide ambience and 95% relative humidity. After 2 h of exposure, the test samples were aspirated and the cells in culture dish were washed three times with phosphate buffer saline to remove any residual nanoemulsion on the cells. The cells were then viewed under confocal electron microscope (Leica, Heidelberg, Germany) for FITC-labeled nanoemulsion that had been undergone intracellular trafficking.

In order to conjugate FITC with chitosan or its folate conjugate, 500 mg of chitosan or chitosan-folate conjugate were dissolved in 100 ml of 0.1 M hydrochloric acid solution (pH adjusted to 3). An accurately weighed 5 mg of FITC was dissolved in 25 ml of methanol, and added to the freshly prepared chitosan or chitosan-folate conjugate solution under continuous magnetic stirring. The reaction between isothiocyanate group of FITC and the primary amino group of d-glucosamine of chitosan was allowed to proceed overnight (24 h) in the dark. The resultant product was coagulated by adding 300 ml of acetone. The coagulate was centrifuged at 3500 rpm for 5 min. The supernatant was discarded and the solid residue was collected. The solid residue was purified by dialysis against distilled water for 24 h, using dialysis membrane (molecular weight cutoff: 1000 Da; Spectra/Por, Spectrum laboratories, Rancho Dominguez, CA). The FITC-bonded chitosan or its folate conjugate was collected and freeze dried at –50 °C for 24 h. The freeze-dried samples were kept in a desiccator till use.

### 2.8. Cytotoxicity

The cytotoxicity of nanoemulsions to normal alveolar macrophages (NR8383) was evaluated by MTT assay. NR8383 were plated in 96 well plate at a cell density of 1 × 10^5^ cells/well and allowed to attach/recover overnight at 5% carbon dioxide ambience and at 37 °C with 95% relative humidity. After 24 h, the medium was removed and 200 µL of the pure drug solution or nanoemulsions were introduced into each well in three different drug concentrations (2.5, 5.0, 10 µg/ml) obtained via serial dilution using serum-free media. Cells without drug solution or nanoemulsions served as the control. The cells were incubated for 24 h in carbon dioxide chamber. Following incubation, the culture media containing pure drug solution or nanoemulsion were removed and 40 µL MTT solution (5 mg/ml in phosphate buffer saline) were added to each well and incubated for an additional 4 h at 37 °C in the dark. Then, the supernatant was aspirated carefully, and the blue formazan crystals formed by reduction of MTT were dissolved in 150 µl of dimethyl sulfoxide with the aid of a microplate shaker (Stuart, Staffordshire, UK). The absorbance was measured at 570 nm on a microplate reader (Sunrise TECAN, Grodig, Austria). At least triplicates were conducted and the results averaged. The cell viability was represented by absorbance ratio of drug solution- or nanoemulsion-treated cells to untreated control, expressed in percentage as follows:

(7) $$\text{Cell viability=}\frac{\text{Absorbance }\text{of }\text{treated }\text{sample}}{\text{Absorbance }\text{of }\text{control}}\times \text{100\%}$$

### 2.9. *In vivo* analysis

Healthy male Sprague–Dawley rats (individual body weight =200 to 250 g; LAFAM, UiTM animal breeding center, Malaysia) were used. They were housed at an ambient temperature of 25 ± 2 °C and a relative humidity of 55 ± 5% on a 12 h light/dark cycle. The rats had free access to standard pelletized food (Gold Coin Enterprise, Malaysia) and de-ionized water *ad libitum*. They were acclimatized for at least 7 days and subjected to 12 h of fasting prior to experiment. All the experiments were approved and conducted according to university ethics regulations adopting the international guidelines (OECD Environment, Health and Safety) of animal experimentation.

#### 2.9.1. Pharmacokinetics analysis

The animals were randomly divided into three groups (*n* = 6/group), and anesthetized by intraperitoneal injection of premixed ketamine and xylazine (15 mg ketamine/200 g rat and 2 mg xylazine/200 g rat). The rats in group 1 were administered with first generation (non-decorated) nanoemulsion, while rats in group 2 and group 3 received second (chitosan-decorated) and third (chitosan-folate conjugate-decorated) generation nanoemulsions, respectively. The samples were administered to the rat lungs at a nominal rifampicin dose of 2 mg/kg using a Microsprayer^®^ aerosolizer (Model IA-C, PennCentury, Inc., Wyndmoor, PA), attached to a high-pressure syringe (PennCentury, Inc.) which was gently inserted through the glottis as described previously (Nahar et al., 2013).

Blood samples (0.2 ml) were collected from tail vein of the rats in heparinized tubes (Reflotron, Roche, Germany) at the intervals of 0.5, 1, 2, 4, 8, 16 and 24 h. The heparinized blood samples were immediately centrifuged at 5000 rpm for 30 min using a tabletop centrifuge (Z216MK, Hermle Labortechnik GmbH, Germany). The plasma was then transferred into microcentrifuge tubes and kept in a freezer at –20 °C until analysis. The plasma drug concentration was examined by first thawing the frozen plasma samples to the ambient temperature. An aliquot of plasma sample was added with methanol at a volume ratio of 1:1 to precipitate the proteins. The mixture was vortexed for 20 min and centrifuged at 5000 rpm for 30 min. The supernatant was isolated, dried under nitrogen gas stream and re-dissolved in 0.5 ml of mobile phase, consisting of an aqueous acetonitrile (pH 2.27 adjusted with orthophosphoric acid) in a volume ratio of acetonitrile to water at 2:3, followed by 5 min vortex and 15 min centrifugation at 5000 rpm. The supernatant was filtered and subjected to HPLC analysis for rifampicin content using the established method as reported by Chaubey & Misra (2014).

Briefly, the HPLC system (Agilent Technologies 1200 series, Agilent, Germany) equipped with C-18 column (Zorbax, 250 × 4.60 mm, particle size 5 µm) and UV-visible detector was used to estimate the drug content. Isocratic elution was operated at the ambient temperature. The flow rate of mobile phase was kept at 1 ml/min and the eluate was monitored at the wavelength of 333 nm. The plasma drug concentrations determined at different time intervals were computed into various pharmacokinetics parameters using PKSolver, an add-in program for pharmacokinetic analysis in Microsoft Excel (Zhang et al., 2010). Calibration curve for drug content was obtained by analyzing blank serum spiked with known amounts of drug.

#### 2.9.2. Drug retention in lung

Three animals of each group were sacrificed by cervical dislocation following 2 h of intratracheal drug administration using Microsprayer^®^ aerosolizer as mentioned above. The lungs were isolated and homogenized with phosphate buffered saline (pH 7.4) and methanol mixture at a volume ratio of 4:1, to extract rifampicin, followed by centrifugation and analysis using the above mentioned HPLC method.

### 2.10. Statistical analysis

Results were expressed as a mean of at least three experiments with the corresponding standard deviation. Statistical data analysis was carried out using SPSS software version 18 (SPSS Inc., Chicago) and a statistically significant difference was denoted by *p* < .05. Student’s-*t*-test and Pearson correlation were employed when applicable.

## 3. Results and discussion

### 3.1. Synthesis and characterization of chitosan-folate conjugate

The chitosan-folate conjugate was prepared according to the method reported by Li et al. (2011). The folate content of conjugate was 12.15 ± 0.44% expressed with respect to the total weight of conjugate and 60.73% of folic acid was conjugated to the chitosan. The conjugate was characterized by FTIR technique. The FTIR spectrum of the conjugate differed from those of chitosan and folic acid, suggesting the formation of chitosan-folate conjugate (Figure 1). The conjugate was characterized by FTIR spectral bands at 3398.00 ± 1.25 cm^−1^ which denoted the O-H/N-H group of chitosan and/or folic acid. Its FTIR peaks at 1614.00 ± 1.27 cm^−1^ and 1521.00 ± 1.60 cm^−1^ exhibited smaller wavenumbers than those of chitosan and folic acid, and this was corresponded to the formation of amide linkage due to chitosan-folate conjugation (Li et al., 2011).

Figure 1.Chemical structures of (a) chitosan, (b) folic acid, (c) chitosan-folate conjugate and their FTIR and NMR spectra.

**Figure:**
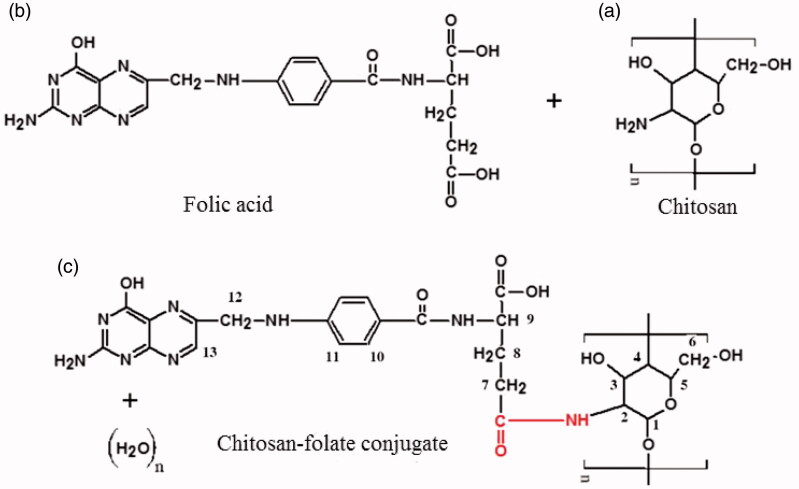

**Figure:**
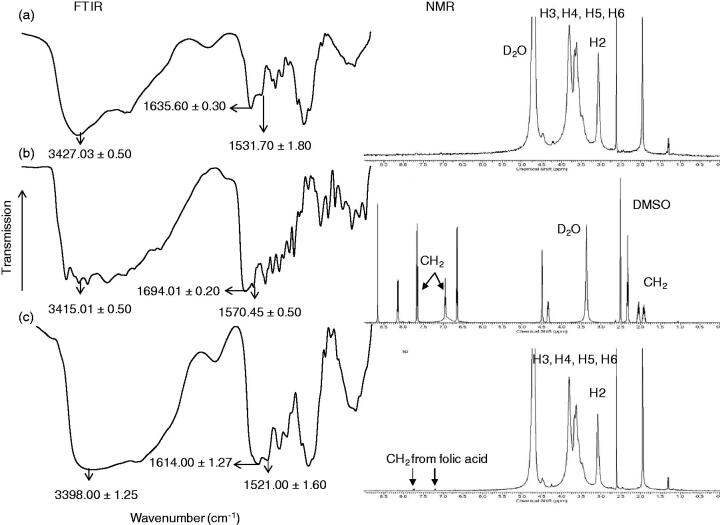

The chitosan-folate conjugate was subjected to analysis by NMR spectroscopy technique. The conjugate gave rise to the characteristic peaks of chitosan at 3.1 to 4.5 ppm ascribing H2, H3, H4, H5 and H6, and a peak at 2.6 ppm which corresponded to the protons from carbon next to the amide moiety, as well as signals at 6.2 to 8.5 ppm corresponding to aromatic protons from folic acid (Figure 1(b)) (Yang et al., 2014). These characteristic peaks in ^1^H-NMR spectrum confirmed that the folic acid was conjugated to chitosan.

### 3.2. Preparation of nanoemulsion

**Figure 2.**
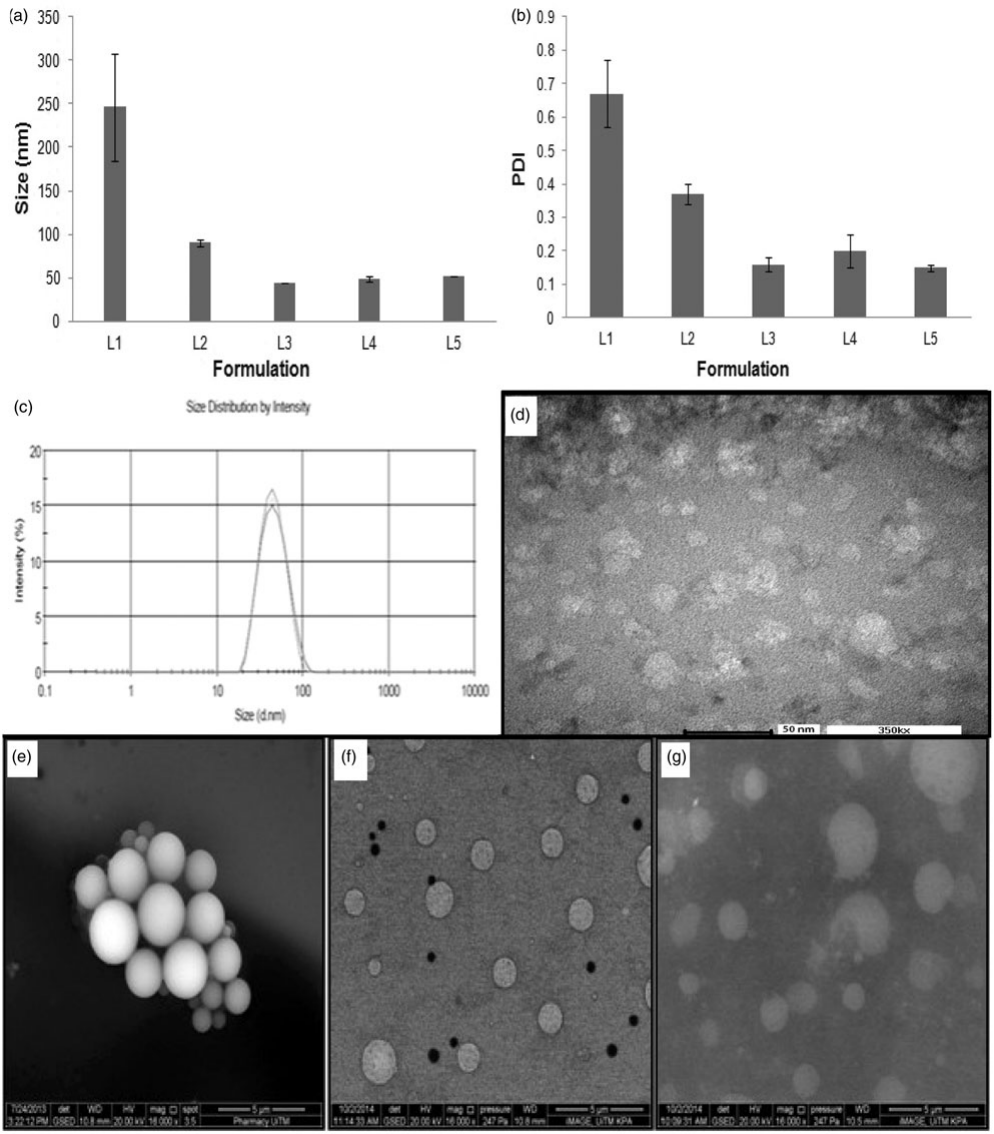
Profiles of (a) average droplet size and (b) polydispersity index of L1 to L5, (c) size distribution and (d) TEM image of L3, ESEM images of (e) first (L3), (f) second (L7) and (g) third (L9) generation nanoemulsions (magnification factor: 16,000 ×).

The nanoemulsion was prepared from pharmaceutically acceptable materials that are generally recognized as safe. Rifampicin was solubilized in oleic acid, in which its maximum drug solubility is 250 ± 1.33 mg/ml (Mehta et al., 2007; Ahmed et al., 2008) and the oil phase was emulsified in normal saline using varying amounts of surfactant and co-solubilizing agent. Photon correlation spectroscopy indicated that the droplet size and polydispersity of the nanoemulsion decreased significantly with an increase in the amount of surfactant used (L1–L3; Table 1, Figure 2(a,b); Student’s-*t*-test: *p* < .05). This was due to adsorption of non-ionic surfactant on the dispersed droplets, enabling a reduction in their surface tension and size through steric stabilization. The mean particle size of 43.89 ± 0.36 nm with polydispersity index at 0.16 ± 0.03 was achievable using 15% Tween 80 in the formulation of first-generation nanoemulsion. Ethanol, when added in concentration above 1% of the nanoemulsion, tended to increase its globule size as well as polydispersity (L3-L5; Table 1, Figure 2(a,b)). This could be due to altered properties of surfactant layers at droplets in the presence of ethanol (Saberi et al., 2013). The ethanol might change the surfactant solubility in the aqueous solution by changing the hydrophobicity of the aqueous phase (Millard et al., 2002) or penetrating into the surfactant monolayer at the oil–water interface and had its optimum curvature, interfacial tension and flexibility altered (Yaghmur et al., 2002). It could also negate the hydration level of the hydrophilic head groups of surfactants, thereby altering the packing and optimum curvature of the surfactant monolayers (Aramaki et al., 1999). In a nutshell, the introduction of ethanol might be associated with a loss of surfactant from the oil–water interface of globules, thereby resulting in an increased globule size and PDI. The first-generation nanoemulsion with 15% Tween 80 and 1% ethanol (L3, Table 1; Figure 2(c,d)) was deemed favorable for use in decoration with targeting ligands for the purpose of alveolar macrophage targeting with the aim to eradicate tubercle bacilli in the course of tuberculosis treatment.

The second generation nanoemulsion was prepared through decorating the droplets of the first-generation nanoemulsion (L3) with chitosan (L6–L8; Table 1). Using 0.25% or 0.5% chitosan, the nanoemulsions produced were characterized by positively charged zeta potential which was deemed favorable in macrophage association via ionic interaction (L6–L8; Table 2(a)). An excessive chitosan fraction, i.e. 0.5%, however, brought about a remarkable increase in the droplet size possibly due to droplet coalescence as a result of binding action of chitosan. This phenomenon reduced the magnitude of the zeta potential instead. The third-generation nanoemulsion (L9; Table 1) was prepared using 0.25% chitosan-folate conjugate instead of chitosan. The chitosan and folate were employed as targeting ligands for mannose and folate receptors located on the membrane surfaces of alveolar macrophages harboring *M*ycobacterium *tuberculosis*.

**Table 2.** Physicochemical characteristics of nanoemulsions with reference to (a) average droplet size, PDI and zeta potential, and (b) pH, surface tension, density, specific viscosity, refractive index, drug content, burst drug release extent and duration to complete drug dissolution.

(a)			
Formulation	Average Size (nm)	PDI	Zeta potential (mV)
L3	43.89 ± 0.36	0.16 ± 0.03	–2.50 ± 1.06
L6	50.70 ± 0.64	0.26 ± 0.03	–1.68 ± 0.12
L7	52.12 ± 0.36	0.25 ± 0.03	4.18 ± 0.13
L8	108.50 ± 0.01	0.46 ± 0.05	2.16 ± 0.50
L9	59.69 ± 0.26	0.23 ± 0.01	0.70 ± 0.24
(b)			
Characteristics	First-generation nanoemulsion (L3)	Second generation nanoemulsion (L7)	Third-generation nanoemulsion (L9)
pH	4.90 ± 0.02	5.06 ± 0.02	4.59 ± 0.04
Surface tension (mN/m^2^)	33.50 ± 0.50	35.00 ± 0.29	36.33 ± 0.94
Density (g/cm^3^)	1.00 ± 0.00	1.01 ± 0.00	1.01 ± 0.00
Specific viscosity	0.16 ± 0.02	0.32 ± 0.01	0.22 ± 0.01
Refractive index	1.34 ± 4.70 × 10^−6^	1.34 ± 4.70 × 10^−6^	1.34 ± 4.70 × 10^−6^
Drug content (mg/ml)	8.51 ± 0.28	8.18 ± 0.35	8.11 ± 0.04
Burst drug release extent (%)	54.82 ± 12.01	38.69 ± 5.71	64.38 ± 12.36
*T*_100%_	6	24	28

Burst drug release extent denoted the fraction of drug released within the first 0.17 h of dissolution.

*T*_100%_ denoted the duration required to achieve 100% drug dissolution.

The addition of chitosan-folate conjugate into the first-generation nanoemulsion raised the size of its droplets (Table 2(a), Student’s-*t*-test: *p* < .05), possibly due to a more expanded structure of conjugate than chitosan alone. The zeta potential of chitosan-decorated nanoemulsion decreased from 4.18 ± 0.13 mV to 0.70 ± 0.24 mV in case of chitosan-folate conjugate-decorated nanoemulsion (Table 2(a), Student’s-*t*-test: *p* < .05), owing to the reduced free amino moieties of chitosan following its conjugation with the folic acid. Broadly, the adsorption of cationic chitosan molecules on the surface of the anionic nanoemulsion droplets caused charge reversal or ‘overcharging’ (Jonsson & Linse, 2001). Overcharging occurred as only a fraction of the positively charged moieties on chitosan was required to neutralize the oppositely charged moieties of oleic acid on the surface of a colloidal particle. The remainder of the charged chitosan moieties could protrude into the aqueous solution or might be in contact with uncharged regions on the globule surfaces (Jonsson & Linse, 2001; Ogawa et al., 2003).

### 3.3. Characterization of nanoemulsion

The first-generation nanoemulsion and its decoration with chitosan or chitosan-folate conjugate could result in changes of nebulization and inhalation profiles as a result of variation in its physicochemical properties.

#### 3.3.1. Morphology

The first-generation nanoemulsion was characterized by spherical dispersed droplets in accordance to the TEM image, with particle size in close agreement to results obtained from the droplet size analysis by means of zetasizer (Figure 2(a,d)). Under the scrutiny of scanning electron microscope, the droplets of the first-generation nanoemulsion were similarly found to be distinctively spherical having smooth surfaces (Figure 2(e)). The second generation nanoemulsion with 0.25% oligochitosan decoration was characterized by relatively well-dispersed droplets (Figure 2(f)). This could be attributed to comparatively high magnitude of zeta potential resulting in repulsion between the dispersed droplets. The surface chitosan could stabilize an emulsion by exerting its steric effect, which generated van der Waal’s repulsion forces between two particles that were too close to one another. The evidence of its presence on the surface of droplets was marked by the rough topical appearance of droplets, demonstrating a higher *R*_a_ value (*R*_a_ = 0.09 ± 0.03 nm for chitosan-decorated nanoemulsion) than those of first-generation nanoemulsion (*R*_a_ = 0.03 ± 0.01). Similar observation was noted with reference to chitosan-folate conjugate-decorated nanoemulsion (Figure 2(g); *R*_a_ = 0.10 ± 0.02 nm).

#### 3.3.2. pH and surface tension

In order to ensure the tolerability of nebulized drugs, an inhaled formulation should have pH values in the range of 4.5–8.7 (Law, 2001). Both ligand-decorated (second and third generation) and non-decorated (first generation) nanoemulsions had pH values between 4.59 and 5.06, fulfilling the specifications for lung administration (Table 2(b)). The addition of chitosan or its conjugate with folate did not alter the pH values of the nanoemulsion significantly (Student’s-*t*-test: *p* > .05).

Surface tension is basically a force experienced by the molecules at the surface and/or at interface of two immiscible liquids. Since alveolar macrophages are located beneath surfactant film on the alveoli surfaces, any changes in the surface tension in the alveolus influence the shape and phagocytic activity of the alveolar macrophages. An increase in the surface tension flattens the alveolar macrophages and decreases their phagocytic activity (Akei et al., 2006). A reduction in the surface tension is expected to improve the interactions of nanoemulsion with alveolar surface and hence phagocytosis. The surface tension values of both ligand-decorated and non-decorated nanoemulsions were lower than those of de-ionized water (71.97 ± 1.22 mN/m^2^) and normal saline (50.61 ± 1.70 mN/m^2^) (Table 2(b)). The reduced surface tension of nanoemulsions was due to the availability of Tween 80, acting as surfactant to decrease the surface tension of the liquid droplets. This decreased surface tension was envisaged to decrease the nebulization time and improve the stability and dispersibility of nanoemulsion. It was expected to aid the contact of nanoemulsion with macrophages and intracellular trafficking of drug.

#### 3.3.3. Density, viscosity and refractive index

The density of a formulation affects the aerodynamic behavior of its nebulized aerosol. In the present investigation, the density of first, second and third-generation nanoemulsions approached unity (Table 2(b)). The viscosity of a nanoemulsion is a function of the surfactant, water and oil components and their respective concentrations. The viscosity of the first-generation nanoemulsion was lower than those of the chitosan- and chitosan-folate conjugate-decorated nanoemulsions (Table 2(b)). The viscosity of the latter was promoted by the presence of polymeric excipients that thickened the droplet interfaces as well as continuous phase. The refractive index of a nanoemulsion is an indicator of its isotropic nature and refractive indices between 1 and 2 represent transparent dosage form. The nanoemulsions had refractive indices close to the value for water (1.334) (Table 2(b)). This suggested that these nanoemulsions possessed homogeneous transparent microstructures.

#### 3.3.4. *In vitro* drug release

**Figure 3.**
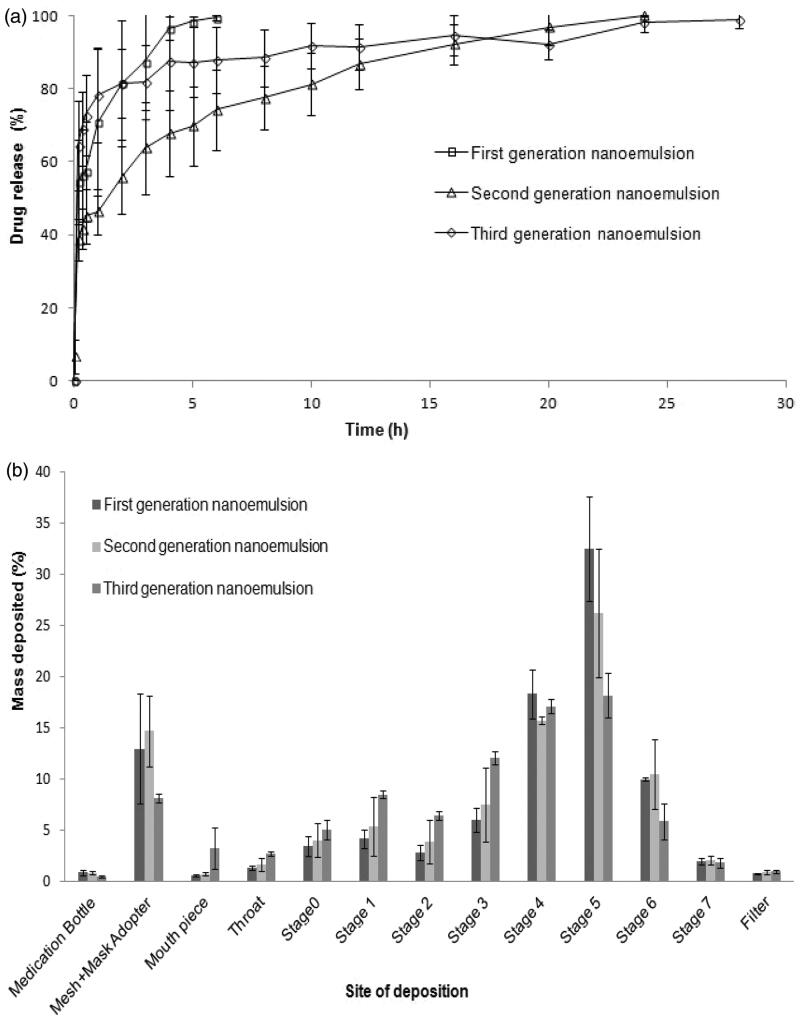
(a) Cumulative percentage drug release profiles and (b) *In vitro* Anderson cascade impaction deposition of drug from first, second and third-generation nanoemulsions.

The nanoemulsion exhibited burst drug release at the early dissolution period, followed by prolonged drug release towards the end of the dissolution phase (Table 2(b); Figure 3(a)). The chitosan-decorated nanoemulsion exhibited the lowest level of burst drug release (Table 2(b); Figure 3(a)) due to the introduction of chitosan into nanoemulsion that raised the viscosity of the liquid and rendered the dispersed droplets less permeable to dissolution medium. The chitosan-folate conjugate-decorated nanoemulsion, unexpectedly, demonstrated the highest level of burst drug release (Table 2(b); Figure 3(a)). This could be due to covalently attaching the folates onto the chitosan chains prevented the polymer chains from aligning themselves at the oil–water interface in an intimate manner. The packing of chitosan-folate conjugate at the oil–water interface was less dense than that of chitosan. This allowed early release of drug embedded near the surfaces of oil droplets. A higher propensity of burst drug release from chitosan-folate conjugate-decorated nanoemulsion was probably due to additional interactive action brought about by folate conjugation of chitosan and this aided a great extent of rifampicin solubilization and dissolution, as suggested by greater viscosity of chitosan-folate conjugate-decorated nanoemulsion than that of first-generation nanoemulsion. Overall, the first-generation nanoemulsion exhibited burst drug release and relatively fast dissolution of drug (Figure 3(a)). The first-generation nanoemulsion (L3) was characterized by small droplet sizes that translated to a large specific area for drug dissolution (Pearson correlation: Size vs. *T*_100%_: *r* = 0.921, *p* = .009). It was relatively less viscous than second (L7) and third (L9) generation nanoemulsions rendering the completion of drug release within a short duration (Table 2(b); Figure 3(a); Pearson correlation: specific viscosity vs. *T*_100%_: *r* = 0.682, *p* = .105). Korsmeyer–Peppas analyzes showed that the release mechanism of nanoemulsions followed Fickian diffusion as marked by *n* values <0.45 (Ritger & Peppas, 1987).

According to the Biopharmaceutical Classification System, rifampicin is class II drug where its bioavailability is dictated by the rate and extent of drug dissolution. Rifampicin shows pH-dependent solubility and dissolution profiles (Agrawal et al., 2004). It dissolves immediately in low-pH media (pH 1.2), but slowly in neutral conditions (Carvalho et al., 2011). This pH-dependent dissolution suggests that the release rate of inhaled rifampicin is potentially retarded in the lung compared to when it is delivered to the gastrointestinal tract. The current *in vitro* drug release findings of nanoemulsions showed an initial burst drug release that could eradicate the non-phagocytosed bacteria in the lungs, while the sustained drug release phase could be beneficial in eradication of phagocytosed bacteria in alveolar macrophages.

#### 3.3.5. Aerodynamic assessment of nebulized aerosol

The aerosolization and inhalation performances of first, second and third-generation nanoemulsions were examined by gravimetric, laser diffraction and cascade impaction methods. The effects of the physicochemical properties of nanoemulsions on their aerosolization and inhalation performances were investigated.

##### 3.3.5.1. Total aerosol output and output rate

In pulmonary drug delivery, the generation of aerosol with an aerodynamic diameter of 1 to 5 µm is a prerequisite to achieve optimum FPF deposition in the deeper lung regions (respiratory bronchioles and alveoli) (Ghazanfari et al., 2007). With reference to nebulization, the aerosol output and aerosol output rate are considered important parameters in order to ensure therapeutic comfort. Typically, the output of aerosolization was higher than 98% (Table 3(a)). The nebulization of first-generation nanoemulsion exhibited higher aerosol output rate than those of the second and third-generation nanoemulsions (Table 3(a); Student’s-*t*-test: *p* < .05). The aerosol output and aerosol output rate were promoted by having a nanoemulsion of lower surface tension together with smaller droplet sizes (Table 3(b); Pearson correlation: Surface tension vs. total aerosol output: *r* = –0.996, *p* = .056; Size vs. total aerosol output: *r* = –0.995, *p* = .064; Surface tension vs. aerosol output rate: *r* = –1, *p* = .010; Size vs. aerosol output rate: *r* = –1, *p* = .017). Our findings were in close agreement with previous studies whereby reducing the droplet size and surface tension of nebulized aerosol promoted efficient aerosolization from the Omron nebulizer (Beck-Broichsitter et al., 2014). The nanoemulsions were formulated with a substantial fraction of normal saline. The high aerosol output could be due to the presence of electrolytes which increased the electrical conductivity and suppressed the electrostatic charge present in pure water or oil droplets. These prevented the oil droplets from adhering to the nebulizer’s surface and mesh holes, and allowed a high fraction of nanoemulsion to be aerosolized.

**Table 3.** Profiling of (a) aerosolization and inhalation performances of nanoemulsions determined using the Anderson cascade impaction and gravimetric methods, and (b) their relationship with the physicochemical properties of nanoemulsions.

(a)Parameter	First-generation nanoemulsion (L3)	Second-generation nanoemulsion (L7)	Third-generation nanoemulsion (L9)		
Total Aerosol Output (%)	99.27 ± 0.41	98.82 ± 0.35	98.53 ± 0.74		
Aerosol output rate (g/min)	0.19 ± 0.01	0.17 ± 0.01	0.15 ± 0.01		
TD (mg)	2.63	2.63	2.63		
ED (mg)	2.49 ± 0.17	2.44 ± 0.09	2.35 ± 0.05		
DD (mg)	2.10 ± 0.20	2.00 ± 0.15	2.00 ± 0.08		
PD (%)	94.68 ± 6.64	93.04 ± 3.62	90.00 ± 1.81		
PI (%)	79.85 ± 7.63	76.04 ± 5.63	75.93 ± 3.18		
MMAD (µm)	3.02 ± 0.19	3.21 ± 0.67	3.84 ± 0.38		
GSD	2.03 ± 0.13	2.12 ± 0.20	2.24 ± 0.02		
FPD _0–f_ (mg)	2.10 ± 0.20	2.00 ± 0.15	1.99 ± 0.08		
FPF	84.38 ± 6.14	81.70 ± 4.47	84.67 ± 2.52		
RF	100.0 ± 0.0	100.0 ± 0.0	100.0 ± 0.0		
FPD_<5µm_ (mg)	1.82 ± 0.22	1.65 ± 0.21	1.47 ± 0.12		
FPF	73.15 ± 5.17	67.71 ± 10.23	62.37 ± 4.58		
RF	86.79 ± 4.52	82.75 ± 10.68	73.60 ± 3.27		
FPD_<3µm_ (mg)	1.66 ± 0.23	1.45 ± 0.31	1.15 ± 0.14		
FPF	66.75 ± 5.31	59.79 ± 14.61	48.88 ± 5.58		
RF	79.26 ± 6.35	72.97 ± 16.58	57.64 ± 4.95		
(b)					
Total aerosol output	*r*	*p*	Aerosol output rate	*r*	*p*
Size	–0.995	.064**	Size	–1.000	.017*
PDI	–0.818	.390	PDI	–0.773	.437
pH	0.549	.630	pH	0.610	.583
Zeta potential	–0.584	.603	Zeta potential	–0.522	.650
Specific viscosity	–0.499	.667	Specific viscosity	–0.434	.714
Surface tension	–0.996	.056**	Surface tension	–1.000	.010*
Refractive index	–0.556	.624	Refractive index	–0.616	.578
PD	*r*	*p*	PI	*r*	*p*
Size	–0.981	.124	Size	–0.889	.302
PDI	–0.625	.571	PDI	–0.975	.144
pH	0.768	.443	pH	0.204	.869
Zeta potential	–0.323	.791	Zeta potential	–0.841	.364
Specific viscosity	–0.225	.855	Specific viscosity	–0.782	.429
Surface tension	–0.979	.131	Surface tension	–0.894	.296
Refractive index	–0.724	.485	Refractive index	–0.139	.911
FPF_<5 µm_	*r*	*P*	FPF_<3 µm_	*r*	*p*
Size	–1.0	.012*	Size	–0.989	.096**
PDI	–0.752	.458	PDI	–0.658	.542
pH	0.643	.555	pH	0.739	.471
Zeta potential	–0.484	.679	Zeta potential	–0.364	.763
Specific viscosity	–0.393	.743	Specific viscosity	–0.268	.827
Surface tension	–1.000	.019*	Surface tension	–0.987	.103
Refractive index	–0.592	.597	Refractive index	–0.693	.513
RF_<5 µm_	*R*	*P*	RF_<3 µm_	*r*	*p*
Size	–0.970	.155	Size	–0.966	.166
PDI	–0.586	.602	PDI	–0.572	.612
pH	0.788	.412	pH	0.808	.401
Zeta potential	–0.276	.822	Zeta potential	–0.26	.833
Specific viscosity	–0.177	.886	Specific viscosity	–0.161	.897
Surface tension	–0.968	.162	Surface tension	–0.963	.173
Refractive index	–0.757	.453	Refractive index	–0.768	.443

Level of significance *p < .050, **p < .100.

##### 3.3.5.2. Cascade impaction analysis

The cascade impaction method, that enabled the aerosol to be characterized as mass of active substance, was applied in the determination of the aerosolization and inhalation patterns of the aerosols. The Omron MicroAir (passive mesh) nebulizer that was developed to overcome the shortcoming of pneumatic and ultrasonic driven devices, such as considerable changes in temperature and concentration of the formulation in reservoir (Beck-Broichsitter et al., 2014), was used in the study. It has been found to have superior performance in nanoemulsion nebulization when compared to standard jet nebulizer (Amani et al., 2010).

Cascade impaction analysis indicated that the first-generation nanoemulsion was characterized by lower MMAD and GSD, generally higher ED, DD, PD, PI, FFD, FPF and RF than those of the second and third-generation nanoemulsions (Table 3(a)). This was attributed at least partially to the smaller droplet sizes and lower surface tension of the former formulation that facilitated its aerosolization and inhalation particularly in the deep lung where FPF_<5 µm_ was concerned (Table 3(b)).

Figure 3(b) showed the *in vitro* deposition pattern of nanoemulsions aerosol subjected to cascade impaction analysis. It has been found that the majority of the nebulized aerosol was deposited on stage 3, 4, 5 and 6, representing aerodynamic cutoff diameters of 4.53, 2.88, 1.51 and 0.96 µm, respectively. This aerosol would be considered to have the most appropriate size range for deep lung deposition by gravitational sedimentation and macrophage targeting. A part of aerosol particles was found to deposit by impaction as suggested by mass fraction collected on stages 0, 1 and 2 with aerodynamic cutoff diameters of 12.36, 7.97 and 6.46 µm, respectively. Together with the mass retained in the throat, this particle fraction represented drug targeted at the upper airways which was crucial in eradication of tubercle bacilli in these regions of the lung.

##### 3.3.5.3. Laser diffraction analysis

The cascade impaction method examined the summative performance of aerosolization and inhalation of nanoemulsions. The laser diffraction method, on the other hand, evaluated the aerosol quality specifically during the stage of aerosolization. It was found that the first-generation nanoemulsion was characterized by smaller droplet size, relatively narrow size distribution, higher FPF_<3 µm_ and FPF_<5 µm_ in accordance to the outcome of the cascade impaction analysis with reference to MMAD, GSD, FPF_<3 µm_ and FPF_<5 µm_ (Tables 3(a) and 4). Unlike cascade impaction analysis, the droplet size and surface tension of a nanoemulsion affected the FPF_<3 µm_ to a greater extent than the FPF_<5 µm_ in an aerosol sample (Pearson correlation: size vs. FPF_<5µm_: *r* = –0.997, *p* = .165; Size vs. FPF_<3µm_: *r* = –0.998, *p* = .036; surface tension vs. FPF_<5µm_: *r* = –0.964, *p* = .172; surface tension vs. FPF_<3µm_: *r* = –0.999, *p* = .029). During the process of cascade impaction, the inhalation of aerosol became more challenging with the lower stages of simulated lung. The influences of droplet size and surface tension of the nanoemulsion on its inhalation profile appeared to be predominated at the deep lung level (FPF_<5 µm_), and to a lesser extent, at the peripheral lung level (FPF_<3 µm_) (Table 3(b)).

**Table 4.** Aerosolization profiles of nanoemulsions determined by laser diffraction method.

Parameter	First generation nanoemulsion (L3)	Second generation nanoemulsion (L7)	Third-generation nanoemulsion (L9)
Dv(10) (µm)	2.33 ± 0.05	2.56 ± 0.07	2.63 ± 0.25
Dv(50) (µm)	4.95 ± 0.07	5.24 ± 0.12	6.34 ± 0.56
Dv(90) (µm)	9.59 ± 0.15	10.28 ± 0.24	14.46 ± 1.83
D[3,2] µm	3.52 ± 0.10	4.49 ± 0.04	4.03 ± 0.12
D[4,3] µm	5.51 ± 0.07	5.93 ± 0.06	7.61 ± 0.32
SSA (m^2^/cc)	1.70 ± 0.04	1.34 ± 0.01	1.49 ± 0.33
GSD/Span	1.87 ± 0.09/1.47 ± 0.01	1.64 ± 0.06/1.47 ± 0.01	2.08 ± 0.08/1.86 ± 0.01
FPF_<3µm_	18.23 ± 0.31	15.09 ± 0.39	12.72 ± 0.87
FPF_<5µm_	45.47 ± 0.59	41.50 ± 0.72	31.88 ± 1.80

#### 3.3.6. Uptake behavior of nanoemulsions by alveolar macrophages

**Figure 4.**
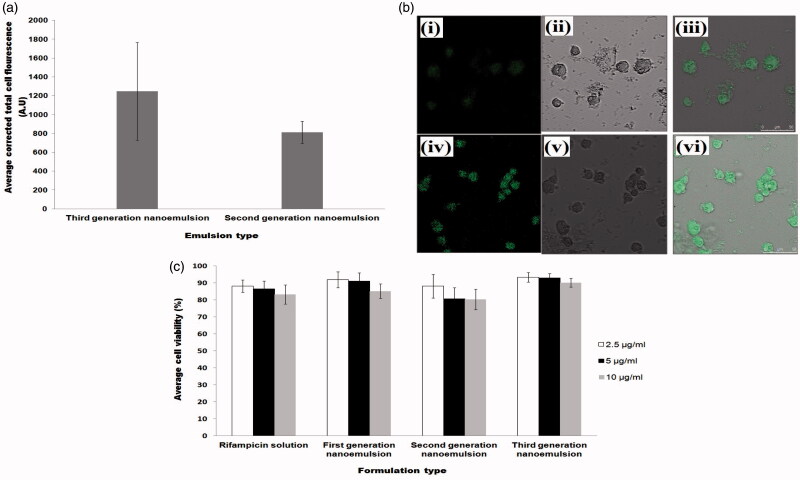
Profiles of (a) average corrected cell fluorescence intensity within alveolar macrophages after treatment with second and third-generation nanoemulsions, (b) confocal laser scanning microscopic images showing endocytosis of second generation nanoemulsion (i–iii) and third-generation nanoemulsion (iv–vi) in macrophages where (i) and (iv) were fluorescent micrographs, (ii) and (v) were differential interference contrast images, (iii) and (vi) were merge images of fluorescent and differential interference contrast images, (c) average viability of alveolar macrophages following treatment with rifampicin solution and rifampicin loaded nanoemulsions.

The internalization of drug loaded carrier by macrophages is crucial for the success of targeted drug delivery in the treatment of tuberculosis. Confocal laser scanning micrographs showed higher intracellular fluorescence intensity in the cytosol of macrophages incubated with nanoemulsions. This was indicative of uptake of nanoemulsion within the cells. A significantly higher level of internalization was observed (Student’s-*t*-test: *p* < .05) with chitosan-folate conjugate-decorated third-generation nanoemulsion when compared to chitosan-decorated second generation nanoemulsion at 2 h of incubation (Figure 4(a,b)). This could be due to the fact that macrophages had over-expressed folate receptors, and cell internalization was facilitated by both folate and mannose receptors mediated endocytosis due to the presence of folate and chitosan moieties in the conjugate. The mannose receptors consists of three extracellular regions, namely, NH_2_-terminal cysteine-rich domain (Cys-MR), C-type lectin carbohydrate-recognition domains and fibronectin II domain (Ezekowitz et al., 1990). Previous studies have shown that glycopolymers containing mannose and N-acetylglucosamine residue can specifically target macrophages in a dose-dependent manner (Song et al., 2012; Chaubey & Mishra, 2014), because of higher expression of mannose receptors on these cells. Similarly, Rollett et al. (2012) had reported that folate receptors are over-expressed on activated macrophages. The chitosan-folate conjugate-decorated third-generation nanoemulsion can be endocytosed with increased affinity due to double receptors targeting.

#### 3.3.7. Cell viability

The nanoemulsions are intended to be used as drug carrier targeting at alveolar macrophages, where mycobacteria are harbored. The incubation of NR8383 cells with drug solutions and nanoemulsions at varying drug concentrations (2.5 µg/ml, 5 µg/ml and 10 µg/ml) for 24 h, did not bring about significant difference in the cell viability of alveolar macrophages (Figure 4(c)). The cell viability was above 80% in general. This signified that the nanoemulsions were safe and biocompatible with reference to rifampicin in therapeutic doses between 2.5 and 10 µg/ml.

#### 3.3.8. Pharmacokinetics

**Figure 5.**
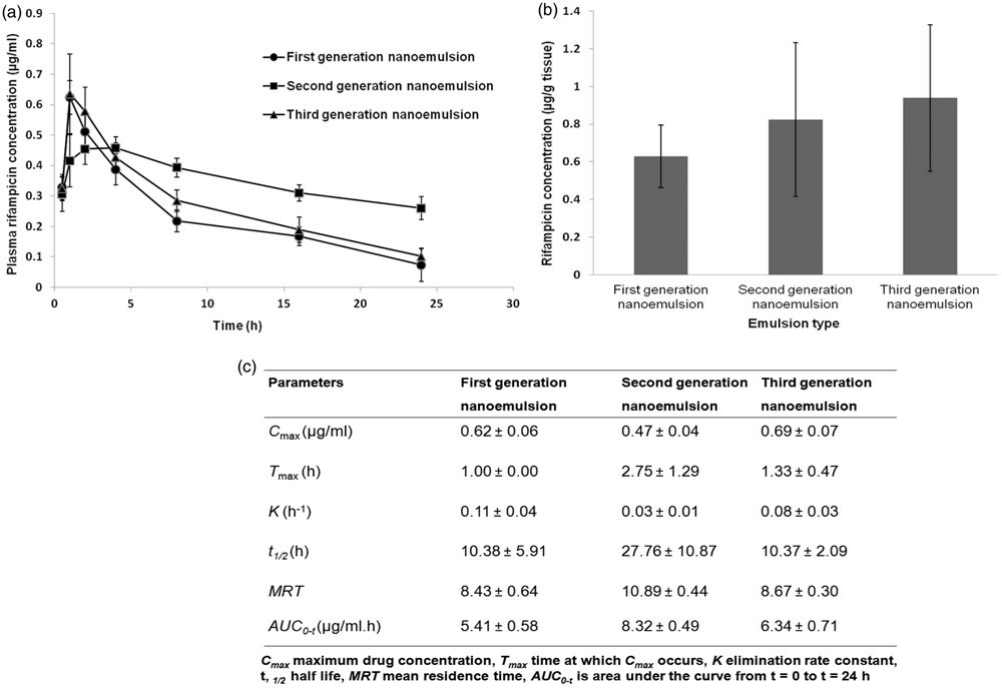
Profiles of (a) average plasma rifampicin concentration-time curves, (b) rifampicin concentrations in homogenized lung and (c) pharmacokinetics parameters obtained after intratracheal administration of rifampicin loaded nanoemulsions at dose of 2 mg/kg body weight.

The average plasma drug concentrations as a function of time after intratracheal administration of the nanoemulsions were shown in Figure 5(a). It was found that the first and third-generation nanoemulsions attained higher plasma concentrations in the first hour when compared to second generation nanoemulsion. This was possibly attributed to higher levels of burst drug release found with first and third-generation nanoemulsions as suggested by the outcome of *in vitro* drug release study (Table 2(b)). Unlike *in vitro* drug release study, the second generation nanoemulsion demonstrated a sustaining plasma drug level *in vivo*, while the third-generation nanoemulsion did not achieve the expectedly high plasma levels of drug at late phase. The latter can be due to a higher level of intracellular trafficking of such nanoemulsion by the alveolar macrophages via double receptor targeting thereby reducing the availability of drug in the plasma (Figure 4(a) and 5). In comparison to first and second generation liquid nanoemulsions, the greater extent of intracellular trafficking of third-generation nanoemulsion by macrophages was reflected by its relatively high lung-drug content (Figure 5(b)).

## 4. Conclusions

In the present study, rifampicin was encapsulated in first generation un-decorated, second generation chitosan-decorated and third generation chitosan-folate conjugate-decorated nanoemulsions for both passive and active drug targeting to macrophages. These nanoemulsions were able to be nebulized with high levels of drug and aerosol output (≥90%). The aerosol output, aerosolized and inhaled FPFs were inversely related to the size and surface tension of the nanoemulsions. The nebulized aerosols were characterized by aerodynamic diameters between 3 and 4 µm, optimum for deep lung deposition as depicted by inhalational FPF_<5 µm_ values between 60 and 75%, and satisfying for peripheral lung deposition and macrophage targeting with FPF_<3 µm_ values ranging from 45 to 70%. All nanoemulsions did not negate the viability of macrophages. The intracellular trafficking of nanoemulsion by macrophages was deemed able to be promoted by chitosan and folate ligands and this led to a high lung drug content of which would be essential for local treatment of tuberculosis.
